# Advances in the mechanism and treatment of mitochondrial quality control involved in myocardial infarction

**DOI:** 10.1111/jcmm.16744

**Published:** 2021-06-23

**Authors:** Chunfang Wang, Leiling Liu, Yishu Wang, Danyan Xu

**Affiliations:** ^1^ Department of Cardiovascular Medicine The Second Xiangya Hospital Central South University Changsha China

**Keywords:** ischemia‐reperfusion, mitochondrial apoptosis, mitochondrial fission, mitochondrial fusion, mitochondrial quality control, myocardial infarction

## Abstract

Mitochondria are important organelles in eukaryotic cells. Normal mitochondrial homeostasis is subject to a strict mitochondrial quality control system, including the strict regulation of mitochondrial production, fission/fusion and mitophagy. The strict and accurate modulation of the mitochondrial quality control system, comprising the mitochondrial fission/fusion, mitophagy and other processes, can ameliorate the myocardial injury of myocardial ischaemia and ischaemia‐reperfusion after myocardial infarction, which plays an important role in myocardial protection after myocardial infarction. Further research into the mechanism will help identify new therapeutic targets and drugs for the treatment of myocardial infarction. This article aims to summarize the recent research regarding the mitochondrial quality control system and its molecular mechanism involved in myocardial infarction, as well as the potential therapeutic targets in the future.

## 1 INTRODUCTION

Myocardial infarction (MI) is generated by myocardial necrosis caused by myocardial hypoxia and myocardial cell energy failure, which are aggravated by long‐term myocardial ischaemia due to coronary occlusion.^1^ Previous studies have shown that abnormal oxidative stress, dysfunction of the antioxidant system and cellular apoptosis play important roles in the occurrence of MI and post‐infarction ischaemia‐reperfusion (I/R).^2^ Mitochondria are the primary sites of energy and ROS production in eukaryotes. Moreover, mitochondria also participate in cell apoptosis and signal transduction. Therefore, normal mitochondrial function is crucial for regulating oxidative stress and apoptosis.^3^

Mitochondria are maintained at a relatively constant state of quantity and function through adaptive remodelling in living cells, termed mitochondrial homeostasis.^4^ Mitochondrial quality control is a significant mechanism for preserving mitochondrial homeostasis and for ensuring the normal function and integrated structure of mitochondria, including mitochondrial dynamics (fission/fusion), autophagy and biogenesis.^5^, ^6^, ^7^ After MI, mitochondria become the first damaged organelle resulting from myocardial hypoxia and reperfusion. The formation of ROS, disruption of the mitochondrial division/fusion balance, abnormal mitophagy, increase in mitochondrial membrane permeability and change in the mitochondrial ultrastructure will induce mitochondrial dysfunction, which is the precursor to the apoptosis of cardiomyocytes. Therefore, stringent quality control of mitochondria is vital for improving and maintaining the normal function of myocardial cells following MI.^8^

## 2 CARDIAC MITOCHONDRIAL DYNAMICS: FISSION/FUSION

Mitochondrial dynamics involve fission and fusion. Mitochondrial fission is the process by which a mitochondrial network structure is broken, forming non‐functional mitochondrial fragments or multiple independent mitochondria. In contrast, mitochondrial fusion is the process by which independent mitochondria or mitochondrial fragments fuse to generate a single mitochondrion.^9^, ^10^, ^11^ With changes to the cell environment, mitochondria continuously split and merge to promote the production of new mitochondria and to repair defective mitochondria. This dynamic process ensures the normal distribution of the mitochondrial metabolites within cells, which is crucial to maintaining the normal function and quality control of mitochondria.^12^

The high expression of mitochondrial fission/fusion‐related proteins in the myocardium suggests that they are essential for modulating cardiomyocyte functionality.^13^ Multiple studies have indicated that when the myocardia is injured by I/R, mitochondrial fission is increased, fusion is inhibited, the balance of fission/fusion is disordered and the structure and function of mitochondria are damaged, thereby impairing the function of myocardial cells.^14^, ^15^, ^16^ Consequently, a precise regulatory mechanism is indispensable for maintaining an equilibrium of fission/fusion, thus ensuring the minimum effective number of mitochondria that can efficiently engage in work.

### 2.1 Mitochondrial Fission of Cardiomyocytes

Mitochondrial fission can increase the number of mitochondria and/or cellular fragility. Fission in cardiomyocytes is primarily mediated by the guanosine triphosphate (GTP) enzyme and dynamin‐related protein 1 (Drp1), which is located in the cytoplasm. It should be noted that the post‐transcriptional modification of Drp1 is very important for mitochondrial fission. Phosphorylation is the main topic. The phosphorylation modification of different sites of Drp1 has reverse effects on mitochondrial fission. For example, cyclin‐dependent kinase 1 (CDK1)/cyclin B (cyclin B) phosphorylates Drp1 S585 (serine 585) and S616(serine 616), promoting mitochondrial division; On the contrary, cyclic AMP‐dependent protein kinase A (PKA) can inhibit mitochondrial fission by phosphorylating Drp1 S656 whereas protein phosphatase dephosphorylates Drp1 S656(serine 656) and S637(serine 637) to promote mitochondrial fission.^17^, ^18^, ^19^, ^20^ In addition, other post‐translational modifications of Drp1, such as ubiquitination, s‐nitrosylation and glycosylation, can also affect mitochondrial fission. Studies have proved that glycosylation of Drp1 facilitates the development of cardiomyopathy, still lacking research on ischaemic heart diseases.^21^, ^22^

**FIGURE 1:**
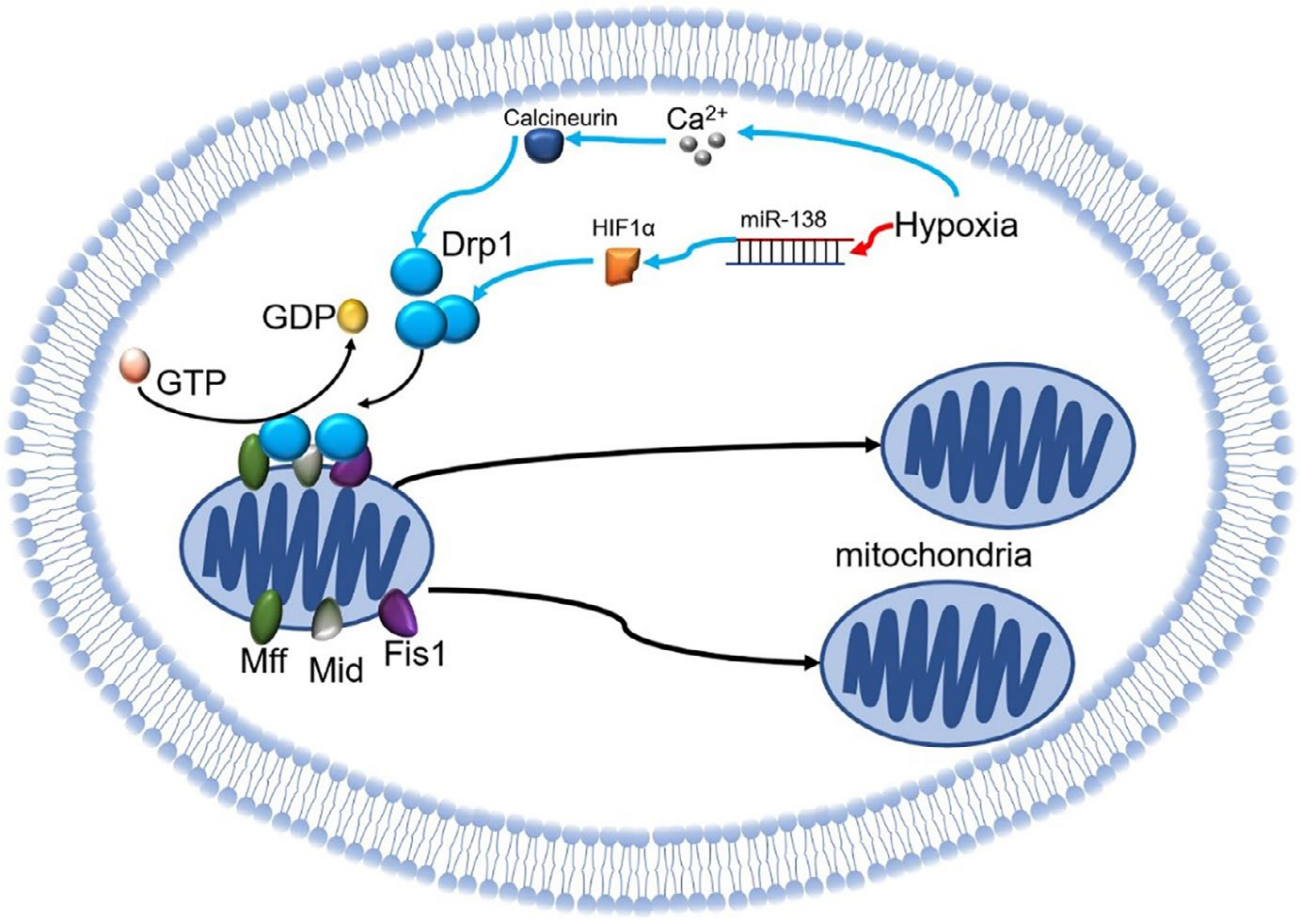
The mechanism of mitochondria fission (Drp1 in the cytoplasm was recruited and translocated to the mitochondria. Drp1 then assembled into a circular polymer by binding with Mid49/51 and Fis 1, Mff on the outer membrane of the mitochondria. This led to the subsequent hydrolysis of GTP and actuated the cleavage of the mitochondrial membrane) and the mechanism of mitochondria fission in MI (red arrow: inactivation; blue arrow: activation; black arrow: direction). (Drp1: dynamin‐related protein 1; Fis1: mitochondrial fission protein 1; GTP: guanosine triphosphate; Mff: mitochondrial fission factor; Mid49/51: mitochondrial dynamic protein 49/51)

A mechanistic study by Otera et al^23^ found that during the course of division, the endoplasmic reticulum was coupled with and wrapped around the mitochondria, whereupon Drp1 in the cytoplasm was recruited and translocated to the mitochondria. Drp1 then assembled into a circular polymer by binding with mitochondrial dynamic protein 49/51 (Mid49/51) and Drp1 receptors including mitochondrial fission protein 1 (Fis 1), mitochondrial fission factor (Mff) on the outer membrane of the mitochondria. This led to the subsequent hydrolysis of GTP and actuated the cleavage of the mitochondrial membrane (Figure 1). Therefore, Drp1‐mediated fission requires the participation of Fis1, Mff and Mid49/51.^8^, ^23^, ^24^, ^25^, ^26^, ^27^ Among them, it was found that Fis1 is not required for mitochondrial fission, whereas Mff plays an important role in the recruitment of Drp1 and Mid49/51 facilitates a connection between Drp1 and Mff on the OMM surface.^23^, ^28^

#### 2.1.1 Mitochondrial Fission after MI

Studies have revealed that hypoxia injury can enhance mitochondrion fission in cardiomyocytes (Figure 1).

Drp1 is an important protein involved in mitochondrial fission. Studies have revealed that the expression and activity of Drp1 are significantly increased after MI, mitochondrial fission of cardiomyocytes is enhanced, the shape of the mitochondria becomes shorter and rounder, and the number of mitochondria rises. This eventually induces an increase in ROS production and cardiomyocyte apoptosis, implying that the abnormal variation of Drp1 after MI may result in an augmentation of mitochondrial fission ^20^, ^29^, ^30^, ^31^, ^32^; In contrast, Ding et al ^33^ found that mitochondrial fission was reduced, the oxidative stress of cardiomyocytes was decreased and the function of mitochondria was improved through drug‐mediated inhibition of Drp1 activity. The above studies demonstrate that the increased Drp1‐mediated mitochondrial fission following MI plays an important pathological role in I/R injury, and the suppression of Drp1 may lessen the degree of myocardial injury.

In a further study of the relevant mechanism by which Drp1 contributes to MI, Sharp et al ^20^ found that calcium overloading in the cytoplasm induced by I/R can activate calcineurin, which accordingly dephosphorylates and then activates Drp1 S637, which is then translocated from the cytoplasm to the outer membrane of mitochondria. This process thereby promotes mitochondrial swelling, cleavage and ROS generation, further aggravating the calcium overload and forms a vicious cycle. On the other hand, Liu et al ^34^ recently found that the expression of microRNA‐138 (miR‐138) in myocardial cells was down‐regulated after MI, which gave rise to the up‐regulation of hypoxia‐inducible factor 1α (HIF‐1α). The increase in HIF‐1α promoted the expression of the mitochondrial division‐related proteins, Drp1 and FIS1, so as to accelerate mitochondrial division. Taken together, the enhancement of Drp1 activity and expression can mediate increased fission, as well as alter the function and morphology of mitochondria.

In addition, regulation of other proteins involved in mitochondrial division may also play an important role. Zhou et al^35^ indicated that the expression level of Mff increased in cardiomyocytes significantly after I/R. Genetic depletion of Mff could reduce the infarct area, ameliorating the vascular endothelial injury and the morphology and function of mitochondria. Samangouei et al^27^ found that after knockout of Mid49/51, the mitochondrial debris of hypoxic cardiomyocytes was decreased, the morphological recovery of mitochondria after reperfusion was accelerated, mitochondrial calcium overload of hypoxic cardiomyocytes was reduced and thus the damage of hypoxic cardiomyocytes was alleviated.^36^

Therefore, a vitally important direction for the treatment of MI may be to seek an appropriate method of inhibiting mitochondrial fission.

#### 2.1.2 Application of MI Therapies

Drp1 represents a pivotal protein in the promotion of mitochondrial fission. Moreover, suppression of the critical process of mitochondrial fission (eg the expression and activity of Drp1, translocation of Drp1 and the binding of Drp1 to regulatory proteins) has been shown to alleviate I/R injury after MI.^16^, ^32^, ^37^, ^38^, ^39^, ^40^, ^41^

Studies have found that inhibiting the expression and activity of Drp1 can improve heart function. For instance, treatment with a P53 inhibitor, PCSK9 inhibitor and melatonin can decrease the level of Drp1 S637 dephosphorylation and facilitate the phosphorylation and deactivation of Drp1, thus suppressing mitochondrial fission.^16^, ^37^, ^39^, ^41^ Secondly, mitochondrial division inhibitor‐1 (Mdivi‐1), a kind of quinazoline derivatives, has been found to reduce the expression of Drp1 and inhibit mitochondrial fission.^14^, ^32^ In addition, Li et al^40^ found that baicalein can up‐regulate the membrane‐associated RING‐CH 5 (MARCH5) in cardiomyocytes via the krüppel‐like factor 4 (KLF4)‐MARCH5‐Drp1 pathway, so as to down‐regulate the level of Drp1 expression in order to relieve I/R injury. As mentioned above, as the down‐regulation of miR‐138 expression can promote cardiomyocyte apoptosis, Liu et al^34^ found a down‐regulation of Drp1 expression in mice and reduction in infarct size following injection with a miR‐138 inhibitor. In conclusion, mitochondrial fission can be weakened by reducing the activity or expression of Drp1 using various inhibitors.

In addition, inhibition of Drp1 translocation to mitochondria and/or its binding to regulatory proteins can also promote heart function. Darwesh et al^42^ found that inhibiting soluble epoxide hydrolase (sEH) can prevent Drp1 from translocating from the cytoplasm to the mitochondria, thus inhibiting mitochondrial fission. As mentioned above, Fis1 cooperates with Drp1 to mediate mitochondria. Disatnik et al^38^ found that P110 can weaken the interaction between Drp1 and Fis1, consequently preventing left ventricular remodelling and reducing the infarct size in rats following MI. Some recent studies have found that the inhibition of Fis1 expression can also inhibit fission.^34^, ^43^ Jin et al^44^ indicated that dual specificity protein phosphatase 1 (DUSP1) can inhibit the activity of JNK, mitigating the binding of JNK and Mff promoter, thus decreasing the expression level of Mff after myocardial infarction and improving mitochondrial and cardiac function. Li et al^45^ found that melatonin can also improve cardiac endothelial function by inhibiting JNK/Mff pathway. Therefore, it can be speculated that the inhibition of other proteins involved in mitochondrial fission may also have a therapeutic effect.

Special attention should be paid to the various risks and limitations associated with suppressive Drp1 therapy. Permanent inhibition of Drp1 activity will have serious consequences. Song et al^46^ found that a knockout of the Drp1 gene can stimulate the opening of mitochondrial permeability transition pore (MPTP) to expedite the release of various apoptosis factors, which induce apoptosis and lead to cardiomyocyte necrosis. In addition, research models of Drp1 are currently primarily limited to small rodents, with little research involving large mammals with inconsistent results. For example, Ong et al^47^ found that there was no cardioprotective effect following Mdivi‐1 therapy in small pigs because there are many affective factors (eg different dosage and administration routes), which require further detailed study.

### 2.2 Mitochondrial Fusion of Cardiomyocytes

Mitochondrial fusion can maintain the normal structure and function of mitochondria together with fission. Fusion relies on mitochondrial transmembrane GTP enzymes, including mitochondrial fusion protein 1 (Mfn1) and mitochondrial fusion protein 2 (Mfn2), which mediate outer mitochondrial membrane (OMM) fusion, as well as optic atrophy 1 (OPA1), which mediates inner mitochondrial membrane (IMM) fusion.^48^

Mitochondrial fusion protein (Mfn, Mfn1 and Mfn2) is a highly conserved transmembrane GTP enzyme encoded by the Mfn gene that is expressed in large quantities in the myocardium. Moreover, Mfn is located in the outer mitochondrial membrane and participates in outer mitochondrial membrane fusion. Chen et al^49^ found that mitochondrial fusion was inhibited after a knockout of the Mfn gene, which resulted in mitochondrial fragmentation and reduced volume, which indicated that Mfn is important in mitochondrial fusion.

OPA1 is a motilin‐related GTP enzyme located in the inner membrane of the mitochondrial crista and participates in the fusion of the mitochondrial inner membrane. Studies have indicated that inhibition of OPA1 expression can lead to significant changes in mitochondrial morphology (eg the rupture of mitochondrial crista structure and the increase in mitochondrial fragmentation phenomenon). At the same time, there is a loss of the mitochondrial membrane potential and a distinct decline in mitochondrial function.^50^ Therefore, OPA1 is also a critical protein involved in mitochondrial fusion and the function.

**FIGURE 2:**
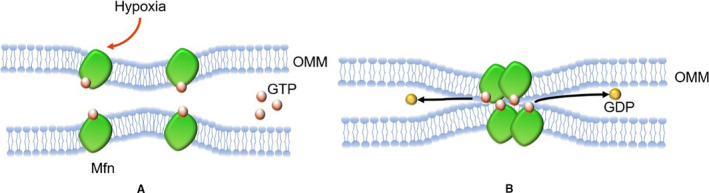
The mechanism of OMM fusion (when two mitochondria are in close contact, the GTP domain of Mfn binds to GTP (A), resulting in the conformational change of Mfn, and followingly GTP hydrolysis promotes the formation of Mfn dimer, thus mediating the OMM fusion (B)) and how MI effects OMM fusion (red arrow: inactivation; black arrow: direction). (GTP: guanosine triphosphate; Mfn: mitochondrial fusion protein; MI: myocardial infarction; OMM: outer mitochondrial membrane)

**FIGURE 3:**
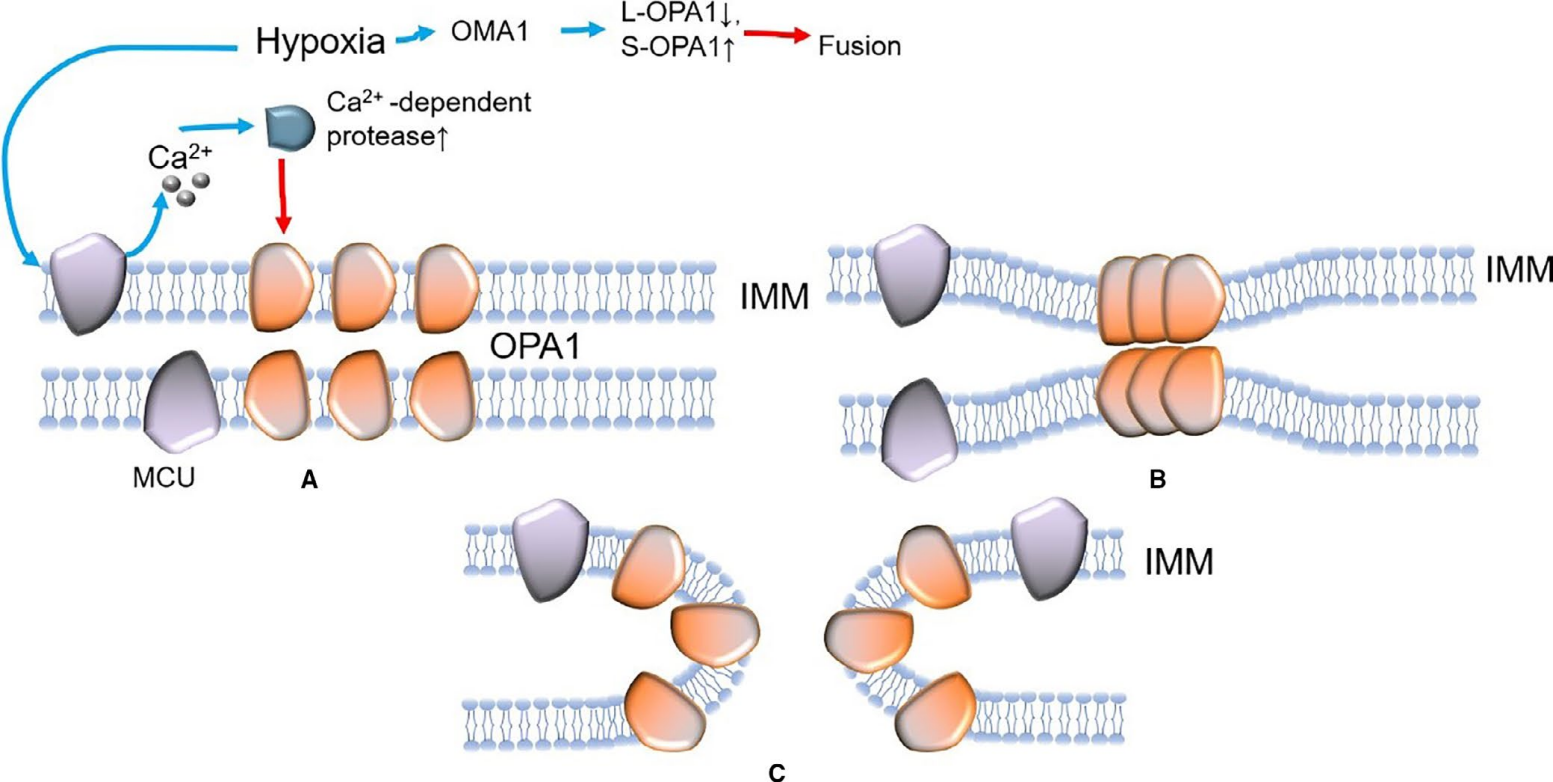
The mechanism of IMM fusion (after OMM fusion, OPA1 forms a polymer Instantaneously to induce IMM curvature (A and B), which leads to lipid fusion of two IMM to form fusion pore, thus mediating IMM fusion (C)) and how MI affects IMM fusion (red arrow: inactivation; blue arrow: activation). (IMM: inner mitochondrial membrane; OPA1: optic atrophy 1)

Mitochondrial fusion of cardiomyocytes can be divided into two steps (Figures 2 and 3): (1) OMM fusion: when two mitochondria are in close contact, the GTP domain of Mfn binds to GTP, resulting in the conformational change of Mfn, and subsequently, GTP hydrolysis promotes the formation of Mfn dimer, thus mediating the OMM fusion; (2) IMM fusion: after OMM fusion, OPA1 forms a polymer instantaneously to induce IMM curvature, which leads to lipid fusion of two IMM to form a fusion pore, thus mediating IMM fusion, and the specific mechanism is still unclear.^51^

#### 2.2.1 Mitochondrial Fusion after MI

Studies have revealed that hypoxia injury can decrease mitochondrion fusion in cardiomyocytes (Figures 2 and 3). Wang et al^52^ found that the structure of the mitochondrial network of myocardial cells was obviously broken, the number of mitochondria increased and the volume decreased in a mouse model of cardiac I/R injury. Studies have found that after MI, the expression of Mfn1 was increased by drug intervention, which promoted mitochondrial fusion and significantly improved mitochondrial function.^53^ Okatan et al^54^ found that I/R caused a substantial decrease in Mfn2 expression, thereby decreasing mitochondrial fusion. These findings suggested that the abnormal expression of Mfn might be related to a decrease in mitochondrial fusion after MI. In contrast, Olmedo et al^55^ found that MG132, a proteasome inhibitor which can inhibit the degradation of Mfn2, or the overexpression of Mfn2 could lessen the broken mitochondrial network of cardiomyocytes and promote mitochondrial fusion.^32^, ^55^ In summary, Mfn is a cardiac protective factor and accelerating the level of Mfn after MI is conducive to improving heart function. However, recent studies indicated that short‐term knockout of Mfn1/2 can increase mitochondrial resistance to MPTP opening during MI, reduce calcium overload and oxidative stress and protect the heart from MI. These studies are contrary to the above results, which may be related to the intervention time of Mfn1/2. Importantly, long‐term depletion of Mfn1/2 is not beneficial to the heart which can lead to cardiac arrest.^56^, ^57^

OPA1, which mediates inner mitochondrial membrane fusion, is abundantly expressed in the heart. After I/R injury, the expression of OPA1 decreases and mitochondrial fusion is also significantly inhibited.^15^, ^43^, ^58^ Guan et al^15^ found that in myocardial I/R injury, the up‐regulated expression of mitochondrial calcium uniporter (MCU) facilitates calcium influx, which causes mitochondrial calcium overload. This results in activation of a calcium‐dependent protease to inhibit OPA1 expression and ultimately leads to decreased mitochondrial fusion. It is suggested that the modulation of mitochondrial fusion by the MCU‐calpain‐OPA1 signalling pathway may be one of the mechanisms of cardiac I/R injury.

In addition, some studies have reflected that the N‐terminal imported peptide of OPA1 can be broken down into a long fragment (L‐OPA1) and a short fragment (S‐OPA1). L‐OPA1 can interact with Mfn1/2 to jointly mediate mitochondrial fusion, whereas S‐OPA1 can inhibit L‐OPA1 function and mitochondrial fusion.^59^, ^60^, ^61^ After MI, the expression of mitochondrial membrane protein 1 (OMA1) increases, which can drive the transformation of L‐OPA1 into S‐OPA1, so as to inhibit the mitochondrial fusion and result in excessive division.^43^, ^62^, ^63^, ^64^, ^65^ It has been further demonstrated that there is increased transformation of L‐OPA1 into S‐OPA1 after MI, which can lead to decreased mitochondrial fusion, which is also an important pathological process of myocardial injury (Figure 3).

In general, the abnormal expression and function of Mfn1/2 and OPA1 play an important role in I/R injury. Therefore, promoting Mfn2 or OPA1 expression and the transformation of OPA1 may represent a potential therapeutic target for MI.

#### 2.2.2 Application in MI Therapies

As mentioned above, Mfn1/2 can be a potential target. Notch1 (a key molecule known to protect heart from IR), mir‐140 inhibitor and long non‐coding RNAs Malat1 can improve mitochondrial membrane potential and protect mitochondrial and heart function by up‐regulating Mfn1 expression.^53^, ^66^, ^67^ Ferreira et al^68^ designed a peptide named SAMβA that selectively antagonizes the association between beta II protein kinase C (βIIPKC) and Mfn1 to deactivate Mfn1, thus attenuating heart failure after MI. Okatan et al^54^ initially found that azoramide can induce an elevation of Mfn2 in the myocardium to promote the fusion of mitochondria after I/R, which ultimately provided cardiac protection. Therefore, azoamine may represent a new regulatory drug for MI treatment. In addition, Maneechote et al^69^ found that the administration of M1, the promoter of the mitochondrial fusion gene, before and after myocardial ischaemia, and after myocardial reperfusion, could up‐regulate the expression of Mfn and reduce the infarct size.

OPA1 may also be a new therapeutic target. Zhang et al^58^ found that an injection of melatonin into the enterocoele before the induction of myocardial I/R in mice can up‐regulate OPA1 expression and enhance mitochondrial fusion after reperfusion injury. Moreover, this protective effect was obviously weakened in OPA1 knockout cells, which indicates that OPA1 is indispensable in the process of melatonin‐regulated mitochondrial fusion. When studying its mechanism, the authors found that I/R can lead to the down‐regulation of Adenosine 5'‐monophosphate (AMP)‐activated protein kinase (AMPK) and OPA1. Melatonin can reverse this effect, and the melatonin‐regulated effect of OPA1 is significantly decreased after inhibiting AMPK. These findings further indicate that melatonin can modulate OPA1 by activating the AMPK signalling pathway. It has been well‐established that melatonin has some important functions (eg scavenging free radicals and antioxidation) and few side effects, which provides insight into novel targets for the development of new drugs for MI.^70^, ^71^ Similarly, irisin, a hormone secreted by muscle, and vitexin, a traditional Chinese medicine that has been used for the treatment of cardiovascular diseases, have all been shown to improve mitochondrial function by increasing the expression of OPA1.^72^, ^73^ In addition, Wang et al^43^ found that 7,8‐dihydroxyflavone(7,8‐DHF), a natural flavonoid, can promote an increase in phosphorylated protein kinase B, which inhibits the OMA1 activity induced by ischaemia and hypoxia. Deactivated OMA1 thus up‐regulates L‐OPA1 and promotes mitochondrial fusion. Therefore, this may also provide a new idea for MI therapy.

The balance of mitochondrial dynamics requires the joint regulation of both fission and fusion. Nevertheless, the mechanism of balance regulation remains unclear yet. Some scholars indicate that fission and fusion are two independent processes, of which their rate is balanced. When one process is down‐regulated, the frequency of another process will increase.^74^ Some studies have found that simultaneous interfering with fusion‐related and fission‐related proteins can maintain mitochondrial homeostasis. For instance, Gao et al^75^ found that C‐Phycocyanin, a type of algae extract, can elevate the expression of OPA1 and Mfn2, and decrease the expression of Drp1 and Fis1 protein, so as to prevent excessive fission of mitochondria. Similarly, Zhao et al^76^ found that continuous short‐term exercise after MI in old mice can inhibit Drp1 expression and increase OPA1 expression, thereby significantly ameliorating the morphology and function of mitochondria. In addition, Naruse et al^77^ found that increasing the expression of GLP‐1 in the intestine after heart failure caused by hypertension can both boost the expression of OPA1 and Mfn1, as well as accelerate the phosphorylation and deactivation of Drp1, subsequently improving mitochondrial function. However, whether the increase in GLP‐1 expression can function in MI by modulating mitochondrial fission and fusion simultaneously still remains to be studied.

In conclusion, the regulation of fusion‐related proteins can ameliorate the apoptosis of myocardial cells after I/R. We speculate that the regulation of other fusion‐related proteins can also be used as therapeutic targets, which requires further exploration.

## 3 MITOPHAGY OF CARDIOMYOCYTES

Mitophagy is the selective removal of damaged or ageing mitochondria through the lysosomal pathway, which is an important process required to maintain mitochondrial homeostasis. This includes cells receiving stimulating signals to form autophagy bodies that specifically recognize mitochondria to be removed. Subsequently, autophagy bodies wrap around abnormal mitochondria and turn to lysosomes to mitochondria. In particular, the specific recognition of mitochondria to be removed by autophagy bodies is a critical step in mitophagy.^78^, ^79^

### 3.1 Mitophagy of Cardiomyocytes

**FIGURE 4:**
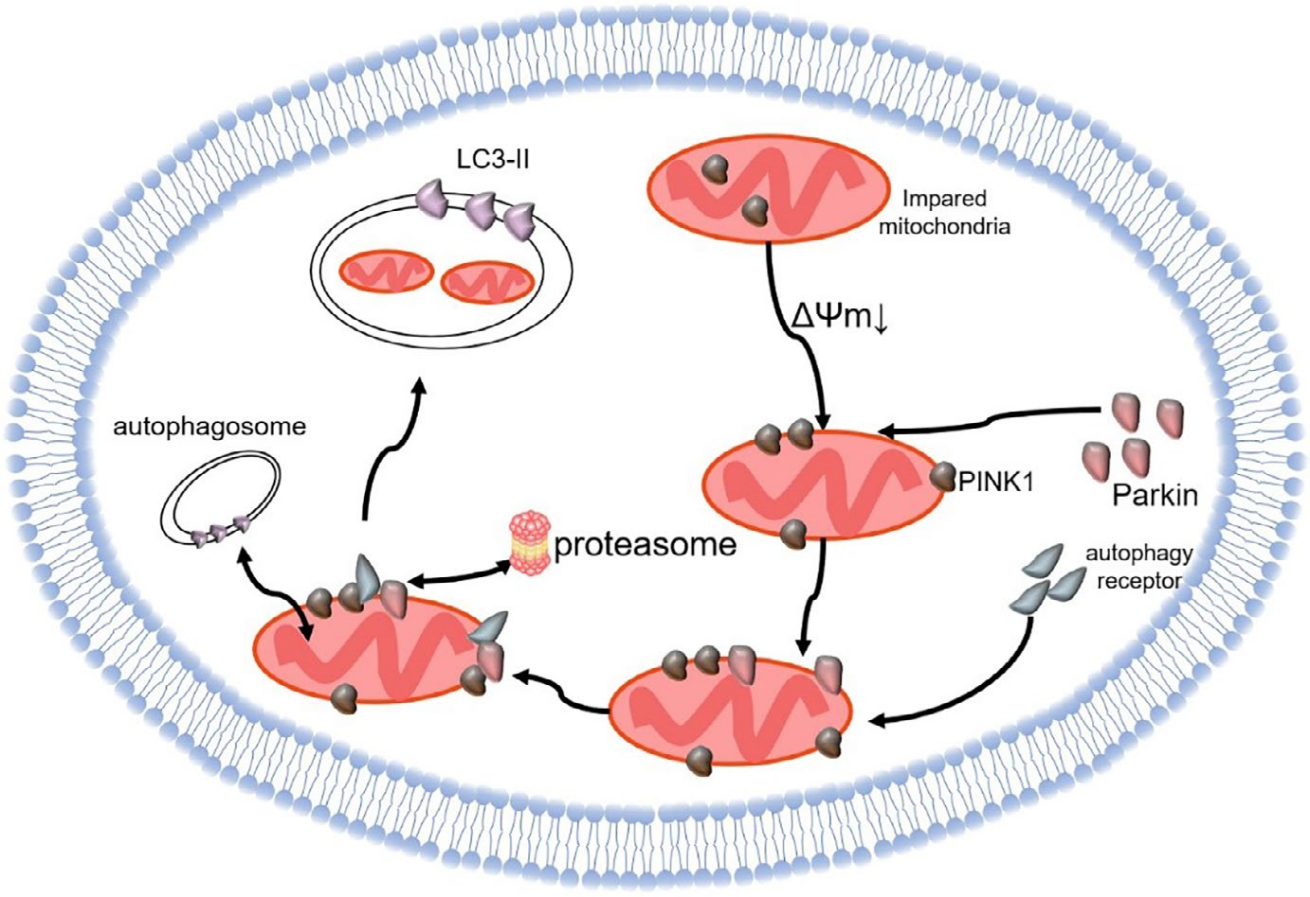
The mechanism of PINK1/Parkin‐dependent mitophagy: the membrane potential of abnormal mitochondria decreased while the expression of PINK1 in OMM increased. PINK1 phosphorylates ubiquitin to activate Parkin, directly collecting autophagy receptor. This is then selectively transferred to damaged mitochondria and mediates the disruption of OMM induced by the proteasome and the degradation of most proteins in outer membrane space Subsequently, IMM protein becomes the target of autophagy through binding with LC3‐II, thus inducing mitophagy.(black arrow: direction). (IMM: inner mitochondrial membrane; LC3‐II: microtube‐associated protein light chain 3‐II; OMM: outer mitochondrial membrane; PINK1: PTEN induced putative kinase 1)

PTEN‐induced putative kinase 1 (PINK1), a serine/threonine‐protein kinase and Parkin, an E3 ubiquitin ligase, are primarily expressed in high energy‐consuming organs, such as the heart and brain.^80^ After mitochondrial damage, the membrane potential decreased whereas the expression of PINK1 in OMM increased. PINK1 phosphorylates ubiquitin to activate Parkin, directly collecting autophagy receptor (eg NDP52) or labelling OMM protein by ubiquitination to collect the autophagosome receptor (eg 62/ SQSTM1). This is then selectively transferred to damaged mitochondria and mediates the disruption of OMM induced by the proteasome and the degradation of most proteins in outer membrane space. Subsequently, IMM protein becomes the target of autophagy through binding with microtube‐associated protein light chain 3‐II (LC3‐II), thus inducing mitophagy. It is suggested that the PINK1/Parkin plays an important role in the removal of damaged mitochondria in normal cells (Figure 4).^81^, ^82^, ^83^

In addition, FUN14 domain‐containing protein 1 (FUNDC1) and Bcl‐2 [B‐cell leukaemia/lymphoma 2]/adenovirus E1B 19KD‐interacting protein 3 (BNIP3) are involved in Parkin‐independent mitophagy. FUNDC1, a type of outer mitochondrial membrane protein, is involved in hypoxia‐induced cardiomyocyte apoptosis and mitophagy.^84^ BNIP3 is a mitochondrial outer membrane protein involved in a variety of pathological processes, such as cardiomyocyte apoptosis and myocardial ischaemia.^85^ FUNDC1 and BNIP3 are mainly involved in mitophagy in pathological states mainly through interaction with LC3‐II.^83^ Respectively, FUNDC1 is phosphorylated and inactivated in mitochondria of normal cells without affecting mitophagy. BNIP3 is also involved in mitochondrial clearance of mature red blood cells.^83^, ^86^

### 3.2 Mitophagy after MI

As mitochondria are the primary sites of ROS production, the opportune removal of abnormal mitochondria can decrease ROS production and provide an energy supplement when suffering from myocardial ischaemia and I/R, which plays an important role in maintaining normal cell growth.^8^

A variety of mitophagy‐related molecules participate in the selective clearance of mitochondria. Thus, regulating these molecules for controlling mitophagy may become an important method for the treatment of MI. Recently, the three most studied pathways are FUNDC1, BNIP3 and PINK1/Parkin.

First, Zhang et al^87^ further found that the autophagy of FUNDC1‐knockout mice was significantly inhibited under hypoxia, suggesting that FUNDC1‐dependent mitophagy plays an important role in hypoxia. Zhou et al^88^ found that reperfusion could phosphorylate and inactivate FUNDC1, suggesting that an inhibition of FUNDC1‐dependent mitophagy at this stage could lead to serious mitochondrial damage. Liu et al^86^ further found that hypoxia could induce the dephosphorylation and activation of FUNDC1 in cardiomyocytes to enhance its interaction withLC3, which was induced by autophagy, thus increasing mitochondrial clearance. Moreover, Yu et al^89^ found that the up‐regulated expression of mammalian STE20‐like kinase 1 (Mst1), a protein kinase, after MI could inhibit the removal of damaged mitochondria by decreasing the activity of FUNDC1. After knocking out the MST1 gene, the infarct size was significantly reduced, which further illustrates that Mst1 is the upstream inhibitor of FUNDC1 and plays an important role in protecting the heart after MI by regulating Mst1 to properly inhibit mitophagy. The above studies show that appropriately enhancing mitophagy after MI and I/R can reduce apoptosis through FUNDC1, which may become potential therapeutic targets of MI.

Second, Lee et al^90^ found that when the level of BNIP3 increased, there was an increase in mitochondrial membrane permeability and mitophagy, suggesting that BNIP3 is correlated with mitophagy. Zhang et al^91^ and Song et al^92^ respectively found that following myocardial I/R, increasing the expression of HIF‐1α or inhibiting the expression of receptor‐interacting protein kinase 3(Ripk3), a cardiac apoptosis‐related programmed necrosis protein, could elevate downstream BNIP3 expression, thereby increasing the clearance rate of damaged mitochondria in cardiomyocytes. These findings provide further indication that BNIP3 mediates mitophagy and modulating HIF‐1α, or that Rip3 can control the activity of BNIP3. Instead, more and more studies have proved that BNIP3 activation or up‐regulation can induce mitochondrial dysfunction. For example, Li et al^93^ indicated that hypoxia‐induced up‐regulation of BNIP3 expression in cardiomyocytes, and the depletion of BNIP3 improved hypoxia‐induced cell damage. Cheng et al^94^ found that BNIP3 knockout can reduce the expression of collagen, ANP, BNP and pro‐apoptotic protein in myocardial tissue of MI.

Third, Zhu et al^95^ found that the phosphorylation level of Parkin decreased after hypoxia injury, whereas overexpression of Parkin can attenuate the opening of MPTP induced by hypoxia, and inhibition of upstream Ripk3 can enhance the phosphorylation of Parkin and improve cardiac function by promoting AMPK phosphorylation. On the contrary, Yao et al^80^ found that I/R can induce the expression of PINK1/Parkin and an increase in mitochondrial autophagy. Some studies have shown that down‐regulating PINK1 and Parkin translocation to mitochondria could attenuate mitophagy and protect cardiac function after I/R.^96^, ^97^

The above studies present different controversial results. The role of mitophagy activation on hypoxic cardiomyocytes is not clear, which brings difficulties to the follow‐up treatment research. It is known that autophagy is a double‐edged sword. Appropriate enhancement of autophagy can protect cells, whereas excessive enhancement or prolonged duration of autophagy can induce apoptosis, leading to tissue necrosis. Nevertheless, how to control the treatment time is a serious problem to be solved. In an article published on *Cell* in 2019, it is proposed that the beneficial effect of autophagy induction is based on MPTP closure and mitochondrial permeability reduction. Although there is a lack of research on the role of mitophagy in MI, we speculate that the effect of autophagy may be related to the cellular or mitochondrial environment. Moreover, BNIP3 has been proved to be related to MPTP opening in mitochondria, which can promote apoptosis, further proving our conjecture.^98^

### 3.3 Application in MI Therapies

Li et al^99^ found that when suffering from myocardial hypoxia, pigment epithelium‐derived factor (PEDF) can up‐regulate FUNDC1 via activating protein kinase C, thereby decreasing the number of damaged mitochondria and increasing the tolerance of myocardial hypoxia. During the reperfusion stage, Zhou et al^88^ found that the inhibition of tyrosine kinase 2α can promote the dephosphorylation and activation of FUNDC1 to accelerate mitophagy and ameliorate cardiomyocyte function. In contrast, Zhou et al^100^ found that melatonin can dephosphorylate and inactivate FUNDC1 to weaken mitophagy through up‐regulating platelet peroxisome proliferator‐activated receptor‐γ (PPAR γ), ultimately reducing the platelet activity and the formation of thrombus after ischaemia and reperfusion. The above studies show that the function of FUNDC1 in MI has a duality in that the activation of FUNDC1 can benefit cardiomyocytes, but harm platelets. This suggests that we should pursue specific regulators for different cell types. For example, we can identify the regulators which target characteristically upstream regulatory signals of FUNDC1 in different cells, so as to improve cell function.

As mentioned above, BNIP3, which was found to be regulated by HIF‐1α and Ripk3, plays an important part in mitophagy. Moreover, Yang et al^101^ found that sevoflurane, an inhaled anaesthetic, can restore BNIP3 expression by up‐regulating HIF‐1α and promote mitophagy before reperfusion treatment in mice with MI. In addition, HIF‐1α selective inhibitors can block this protective effect, which provides a new option for preoperative anaesthetic with MI. Similarly, Liu et al^102^ found that panax notoginseng saponins, a type of Chinese medicine that has been used for the clinical treatment of MI, can also protect the heart by up‐regulating HIF‐1α/BNIP3. On the other hand, Shin et al^103^ found that after transfecting microRNA‐105 into myocardial I/R cells, microRNA‐105 could be coupled with Rip3 and BNIP3 mRNA, finally increasing BNIP3 expression and decreasing apoptosis. On the contrary, Jin et al^44^ found that DUSP1 could inhibit BNIP3 phosphorylation by inhibiting JNK pathway, thus improving mitochondrial function. Yeang et al^104^ found that E06, an antioxidant phospholipid antibody, can inhibit the expression of BNIP3 and reduce the infarct size after I/R by inhibiting oxidized phospholipids. Other drugs such as curcumin and dihydromyricetin also play the same role.^105^, ^106^ In conclusion, BNIP3‐mediated mitophagy has completely different effects.

PINK/Parkin can mediate mitophagy during MI. It has been found that cardiac resynchronization therapy following heart failure caused by MI and berberine, which belongs to the traditional Chinese medicine, can all induce an increase in PINK/Parkin expression and mitophagy.^107^, ^108^ Yao et al^80^ found that an injection of hydrogen‐rich saline can attenuate inflammation and apoptosis of the damaged myocardium by enhancing mitophagy. The authors further found that after knocking out the PINK gene, the protective effect of hydrogen was partially reversed, suggesting that hydrogen can enhance PINK/Parkin‐mediated mitophagy to attenuate MI. Particularly, Zhao et al^76^ further found that proper exercise can ascend the expression of PINK1/Parkin and accelerate the removal of damaged mitochondria after MI; however, this effect is significantly related to the time and frequency of exercise. Differing amounts of exercise may have various effects on mitophagy and the outcome of diseases. An excessive amount of exercise may lead to mitochondrial dysfunction and the attenuation of mitochondrial function by short‐term exercise is not permanent, which suggests that exercise programmes should be reasonably formulated in clinical treatment.^76^, ^109^, ^110^, ^111^, ^112^, ^113^ Besides, Ferulic acid or G protein‐coupled oestrogen receptor 1 (GPER) can protect cardiac function after I/R by down‐regulating PINK1 and Parkin translocation to mitochondria and reducing mitophagy,^96^, ^97^ which gives us a problem again that how to control mitophagy via Parkin.

Modulating mitophagy and selectively targeting the heart may represent a new direction for the treatment of MI. In addition, some traditional Chinese medicine that has been proven to regulate mitophagy (eg berberine and panax notoginseng saponins) has been used clinically,^108^, ^114^ and other clinically relevant drugs are still under study. East et al^115^ summarized that some compounds, including carbonyl cyanide m‐chlorophenylhydrazone and antimycin/oligomycin, can non‐specifically induce mitophagy; however, they have a high degree of toxicity and thus cannot be applied in the clinical treatment of MI. In addition, the authors discovered a mitophagy inducer (PMI), which could promote the mitophagy of both nerve and liver cells; however, there has not been any therapeutic research in an MI model.^116^ Notably, there are still controversies about the regulation of mitophagy in the heart. The role of BNIP3 in regulating mitophagy in MI remains exclusive. This kind of mitophagy may occur in a specific environment and at a specific time, and BNIP3 has a significant role in promoting apoptosis. More studies believe that inhibition of BNIP3 will bring beneficial effects. This makes it more difficult to treat MI with BNIP3 as the target. More experiments may be needed to prove which regulation is dominant after MI and cardiac I/R.^117^

## 4 CONCLUSION

Increased attention has been paid to the role of mitochondrial quality control in MI, which provides a variety of therapeutic targets for the treatment of MI, whereas all aspects of mitochondrial quality control in MI, such as mitochondrial fission/fusion and mitophagy, are not isolated. For example, mitochondrial fission/fusion and mitophagy are interrelated. Mitochondrial fission is significantly increased after MI. Twig et al^64^ found that mitochondrial fission can produce non‐functional depolarized mitochondria, of which the morphology was changed, membrane potential was lost, the OPA1 expression was decreased and the fusion ability was significantly weakened, finally becoming the target of mitophagy. In general, fission can promote mitophagy and remove depolarized mitochondria. In addition, the fusion ability of target mitochondria is significantly weakened before autophagy, which suggests that various processes of mitochondrial quality control should be considered, rather than treating them as an isolated unit, providing us with new ideas for the treatment of MI. However, there are currently no specific drugs that have been found for clinical treatment, and further experiments are needed to identify novel drugs.

## CONFLICT OF INTEREST

No conflict of interest was declared.

## AUTHOR CONTRIBUTION

**Chunfang Wang**: Conceptualization (lead); Supervision (lead); Writing‐original draft (lead); Writing‐review & editing (lead). **Leiling Liu**: Writing‐review & editing (supporting). **Yishu Wang**: Writing‐review & editing (supporting). **Danyan Xu**: Supervision (lead); Validation (lead).

## Data Availability

Not applicable.

## References

[1] Tibaut M , Mekis D , Petrovic D . Pathophysiology of myocardial infarction and acute management strategies. Cardiovasc Hematol Agents Med Chem. 2017;14(3):150‐159. 27993119 10.2174/1871525714666161216100553

[2] Yue R , Xia X , Jiang J , et al. Mitochondrial DNA oxidative damage contributes to cardiomyocyte ischemia/reperfusion‐injury in rats: cardioprotective role of lycopene. J Cell Physiol. 2015;230(9):2128‐2141. 25656550 10.1002/jcp.24941

[3] Crack PJ , Taylor JM . Reactive oxygen species and the modulation of stroke. Free Radic Biol Med. 2005;38(11):1433‐1444. 15890617 10.1016/j.freeradbiomed.2005.01.019

[4] Lyons J , Rauh‐Pfeiffer A , Ming‐Yu Y , et al. Cysteine metabolism and whole blood glutathione synthesis in septic pediatric patients. Crit Care Med. 2001;29(4):870‐877. 11373484 10.1097/00003246-200104000-00036

[5] Lan B , He Y , Sun H , Zheng X , Gao Y , Li N . The roles of mitochondria‐associated membranes in mitochondrial quality control under endoplasmic reticulum stress. Life Sci. 2019;231:116587. 31220526 10.1016/j.lfs.2019.116587

[6] Liu H , Li S , Liu X , Chen Y , Deng H . SIRT3 overexpression inhibits growth of kidney tumor cells and enhances mitochondrial biogenesis. J Proteome Res. 2018;17(9):3143‐3152. 30095923 10.1021/acs.jproteome.8b00260

[7] Samant SA , Zhang HJ , Hong Z , et al. SIRT3 deacetylates and activates OPA1 to regulate mitochondrial dynamics during stress. Mol Cell Biol. 2014;34(5):807‐819. 24344202 10.1128/MCB.01483-13 PMC4023816

[8] Anzell AR , Maizy R , Przyklenk K , Sanderson TH . Mitochondrial quality control and disease: Insights into Ischemia‐Reperfusion injury. Mol Neurobiol. 2018;55(3):2547‐2564. 28401475 10.1007/s12035-017-0503-9 PMC5636654

[9] Chan DC . Mitochondrial fusion and fission in mammals. Annu Rev Cell Dev Biol. 2006;22:79‐99. 16704336 10.1146/annurev.cellbio.22.010305.104638

[10] Dorn GW 2nd . Evolving concepts of mitochondrial dynamics. Annu Rev Physiol. 2019;81:1‐17. 30256725 10.1146/annurev-physiol-020518-114358

[11] Zhang D , Ma J . Mitochondrial dynamics in rat heart induced by 5‐Fluorouracil. Med Sci Monit. 2018;24:6666‐6672. 30240386 10.12659/MSM.910537 PMC6228119

[12] Knott AB , Perkins G , Schwarzenbacher R , Bossy‐Wetzel E . Mitochondrial fragmentation in neurodegeneration. Nat Rev Neurosci. 2008;9(7):505‐518. 18568013 10.1038/nrn2417 PMC2711514

[13] Hom J , Sheu SS . Morphological dynamics of mitochondria–a special emphasis on cardiac muscle cells. J Mol Cell Cardiol. 2009;46(6):811‐820. 19281816 10.1016/j.yjmcc.2009.02.023 PMC2995918

[14] Ding M , Dong Q , Liu Z , et al. Inhibition of dynamin‐related protein 1 protects against myocardial ischemia‐reperfusion injury in diabetic mice. Cardiovasc Diabetol. 2017;16(1):19. 28173848 10.1186/s12933-017-0501-2 PMC5297196

[15] Guan L , Che Z , Meng X , et al. MCU Up‐regulation contributes to myocardial ischemia‐reperfusion Injury through calpain/OPA‐1‐mediated mitochondrial fusion/mitophagy Inhibition. J Cell Mol Med. 2019;23(11):7830‐7843. 31502361 10.1111/jcmm.14662 PMC6815825

[16] Dube K , Dhanabalan K , Salie R , Blignaut M , Huisamen B , Lochner A . Melatonin has profound effects on mitochondrial dynamics in myocardial ischaemia/reperfusion. Heliyon. 2019;5(10):e02659. 31720456 10.1016/j.heliyon.2019.e02659 PMC6838907

[17] Cribbs JT , Strack S . Reversible phosphorylation of Drp1 by cyclic AMP‐dependent protein kinase and calcineurin regulates mitochondrial fission and cell death. EMBO Rep. 2007;8(10):939‐944. 17721437 10.1038/sj.embor.7401062 PMC2002551

[18] Taguchi N , Ishihara N , Jofuku A , Oka T , Mihara K . Mitotic phosphorylation of dynamin‐related GTPase Drp1 participates in mitochondrial fission. J Biol Chem. 2007;282(15):11521‐11529. 17301055 10.1074/jbc.M607279200

[19] Cereghetti GM , Stangherlin A , de Brito OM , et al. Dephosphorylation by calcineurin regulates translocation of Drp1 to mitochondria. Proc Natl Acad Sci USA. 2008;105(41):15803‐15808. 18838687 10.1073/pnas.0808249105 PMC2572940

[20] Sharp WW , Fang YH , Han M , et al. Dynamin‐related protein 1 (Drp1)‐mediated diastolic dysfunction in myocardial ischemia‐reperfusion injury: therapeutic benefits of Drp1 inhibition to reduce mitochondrial fission. FASEB J. 2014;28(1):316‐326. 24076965 10.1096/fj.12-226225 PMC3868827

[21] Adaniya SM , O‐Uchi J , Cypress MW , Kusakari Y , Jhun BS . Posttranslational modifications of mitochondrial fission and fusion proteins in cardiac physiology and pathophysiology. Am J Physiol Cell Physiol. 2019;316(5):C583‐C604. 30758993 10.1152/ajpcell.00523.2018 PMC6580160

[22] Jhun B , O‐Uchi J , Adaniya S , Cypress M , Yoon Y . Adrenergic regulation of Drp1‐Driven mitochondrial fission in cardiac physio‐pathology. Antioxidants (Basel). 2018;7(12):195. 10.3390/antiox7120195 PMC6316402 30567380

[23] Otera H , Wang C , Cleland MM , et al. Mff is an essential factor for mitochondrial recruitment of Drp1 during mitochondrial fission in mammalian cells. J Cell Biol. 2010;191(6):1141‐1158. 21149567 10.1083/jcb.201007152 PMC3002033

[24] Zhao J , Liu T , Jin S , et al. Human MIEF1 recruits Drp1 to mitochondrial outer membranes and promotes mitochondrial fusion rather than fission. EMBO J. 2011;30(14):2762‐2778. 21701560 10.1038/emboj.2011.198 PMC3160255

[25] Palmer CS , Osellame LD , Laine D , Koutsopoulos OS , Frazier AE , Ryan MT . MiD49 and MiD51, new components of the mitochondrial fission machinery. EMBO Rep. 2011;12(6):565‐573. 21508961 10.1038/embor.2011.54 PMC3128275

[26] Elgass KD , Smith EA , LeGros MA , Larabell CA , Ryan MT . Analysis of ER‐mitochondria contacts using correlative fluorescence microscopy and soft X‐ray tomography of mammalian cells. J Cell Sci. 2015;128(15):2795‐2804. 26101352 10.1242/jcs.169136 PMC4540952

[27] Samangouei P , Crespo‐Avilan GE , Cabrera‐Fuentes H , et al. MiD49 and MiD51: New mediators of mitochondrial fission and novel targets for cardioprotection. Cond Med. 2018;1(5):239‐246. 30338314 PMC6191188

[28] Yu R , Liu T , Jin S‐B , et al. MIEF1/2 function as adaptors to recruit Drp1 to mitochondria and regulate the association of Drp1 with Mff. Sci Rep. 2017;7(1):880. 28408736 10.1038/s41598-017-00853-x PMC5429825

[29] Zepeda R , Kuzmicic J , Parra V , et al. Drp1 loss‐of‐function reduces cardiomyocyte oxygen dependence protecting the heart from ischemia‐reperfusion injury. J Cardiovasc Pharmacol. 2014;63(6):477‐487. 24477044 10.1097/FJC.0000000000000071

[30] Luo H , Song S , Chen Y , et al. Inhibitor 1 of Protein Phosphatase 1 Regulates Ca(2+)/Calmodulin‐Dependent Protein Kinase II to Alleviate Oxidative Stress in Hypoxia‐Reoxygenation Injury of Cardiomyocytes. Oxid Med Cell Longev. 2019;2019:2193019. 31885777 10.1155/2019/2193019 PMC6925801

[31] Willems PH , Rossignol R , Dieteren CE , Murphy MP , Koopman WJ . Redox homeostasis and mitochondrial dynamics. Cell Metab. 2015;22(2):207‐218. 26166745 10.1016/j.cmet.2015.06.006

[32] Ong S‐B , Subrayan S , Lim SY , Yellon DM , Davidson SM , Hausenloy DJ . Inhibiting mitochondrial fission protects the heart against ischemia/reperfusion injury. Circulation. 2010;121(18):2012‐2022. 20421521 10.1161/CIRCULATIONAHA.109.906610

[33] Ding M , Dong Q , Liu Z , et al. Erratum to: Inhibition of dynamin‐related protein 1 protects against myocardial ischemia‐reperfusion injury in diabetic mice. Cardiovasc Diabetol. 2017;16(1):60. 28472959 10.1186/s12933-017-0540-8 PMC5418774

[34] Liu Y , Zou J , Liu X , Zhang Q . MicroRNA‐138 attenuates myocardial ischemia reperfusion injury through inhibiting mitochondria‐mediated apoptosis by targeting HIF1‐alpha. Exp Ther Med. 2019;18(5):3325‐3332. 31602205 10.3892/etm.2019.7976 PMC6777330

[35] Zhou H , Hu S , Jin Q , et al. Mff‐Dependent mitochondrial fission contributes to the pathogenesis of cardiac microvasculature ischemia/reperfusion injury via induction of mROS‐Mediated cardiolipin oxidation and HK2/VDAC1 disassociation‐involved mPTP opening. J Am Heart Assoc. 2017;6(3):e005328. 28288978 10.1161/JAHA.116.005328 PMC5524036

[36] Atkins K , Dasgupta A , Chen K‐H , Mewburn J , Archer SL . The role of Drp1 adaptor proteins MiD49 and MiD51 in mitochondrial fission: implications for human disease. Clin Sci (Lond). 2016;130(21):1861‐1874. 27660309 10.1042/CS20160030

[37] Din S , Mason M , Völkers M , et al. Pim‐1 preserves mitochondrial morphology by inhibiting dynamin‐related protein 1 translocation. Proc Natl Acad Sci USA. 2013;110(15):5969‐5974. 23530233 10.1073/pnas.1213294110 PMC3625351

[38] Disatnik M‐H , Ferreira JCB , Campos JC , et al. Acute inhibition of excessive mitochondrial fission after myocardial infarction prevents long‐term cardiac dysfunction. J Am Heart Assoc. 2013;2(5):e000461. 24103571 10.1161/JAHA.113.000461 PMC3835263

[39] Wang J‐X , Jiao J‐Q , Li Q , et al. miR‐499 regulates mitochondrial dynamics by targeting calcineurin and dynamin‐related protein‐1. Nat Med. 2011;17(1):71‐78. 21186368 10.1038/nm.2282

[40] Li QI , Yu Z , Xiao D , et al. Baicalein inhibits mitochondrial apoptosis induced by oxidative stress in cardiomyocytes by stabilizing MARCH5 expression. J Cell Mol Med. 2020;24(2):2040‐2051. 31880404 10.1111/jcmm.14903 PMC6991701

[41] Palee S , McSweeney CM , Maneechote C , et al. PCSK9 inhibitor improves cardiac function and reduces infarct size in rats with ischaemia/reperfusion injury: Benefits beyond lipid‐lowering effects. J Cell Mol Med. 2019;23(11):7310‐7319. 31557388 10.1111/jcmm.14586 PMC6815840

[42] Darwesh AM , Keshavarz‐Bahaghighat H , Jamieson KL , Seubert JM . Genetic deletion or pharmacological inhibition of soluble epoxide hydrolase ameliorates cardiac ischemia/reperfusion injury by attenuating NLRP3 inflammasome activation. Int J Mol Sci. 2019;20(14):3502. 10.3390/ijms20143502 PMC6678157 31319469

[43] Wang Z , Wang S‐P , Shao Q , et al. Brain‐derived neurotrophic factor mimetic, 7,8‐dihydroxyflavone, protects against myocardial ischemia by rebalancing optic atrophy 1 processing. Free Radic Biol Med. 2019;145:187‐197. 31574344 10.1016/j.freeradbiomed.2019.09.033

[44] Jin Q , Li R , Hu N , et al. DUSP1 alleviates cardiac ischemia/reperfusion injury by suppressing the Mff‐required mitochondrial fission and Bnip3‐related mitophagy via the JNK pathways. Redox Biol. 2018;14:576‐587. 29149759 10.1016/j.redox.2017.11.004 PMC5691221

[45] Li P , Xie C , Zhong J , Guo Z , Guo K , Tu Q . Melatonin attenuates ox‐LDL‐Induced endothelial dysfunction by reducing ER stress and inhibiting JNK/Mff signaling. Oxid Med Cell Longev. 2021;2021:5589612. 33763168 10.1155/2021/5589612 PMC7952160

[46] Song M , Mihara K , Chen Y , Scorrano L , Dorn GW . Mitochondrial fission and fusion factors reciprocally orchestrate mitophagic culling in mouse hearts and cultured fibroblasts. Cell Metab. 2015;21(2):273‐286. 25600785 10.1016/j.cmet.2014.12.011 PMC4318753

[47] Ong S‐B , Kwek X‐Y , Katwadi K , et al. Targeting mitochondrial fission using Mdivi‐1 in A clinically relevant large animal model of acute myocardial infarction: a pilot study. Int J Mol Sci. 2019;20(16):3972. 10.3390/ijms20163972 PMC6720595 31443187

[48] Meyer JN , Leuthner TC , Luz AL . Mitochondrial fusion, fission, and mitochondrial toxicity. Toxicology. 2017;391:42‐53. 28789970 10.1016/j.tox.2017.07.019 PMC5681418

[49] Chen H , Detmer SA , Ewald AJ , Griffin EE , Fraser SE , Chan DC . Mitofusins Mfn1 and Mfn2 coordinately regulate mitochondrial fusion and are essential for embryonic development. J Cell Biol. 2003;160(2):189‐200. 12527753 10.1083/jcb.200211046 PMC2172648

[50] Olichon A , Baricault L , Gas N , et al. Loss of OPA1 perturbates the mitochondrial inner membrane structure and integrity, leading to cytochrome c release and apoptosis. J Biol Chem. 2003;278(10):7743‐7746. 12509422 10.1074/jbc.C200677200

[51] Gao S , Hu J . Mitochondrial fusion: The machineries in and out. Trends Cell Biol. 2021;31(1):62‐74. 33092941 10.1016/j.tcb.2020.09.008

[52] Wang K , Liu Z , Zhao M , et al. kappa‐opioid receptor activation promotes mitochondrial fusion and enhances myocardial resistance to ischemia and reperfusion injury via STAT3‐OPA1 pathway. Eur J Pharmacol. 2020;874:172987. 32032598 10.1016/j.ejphar.2020.172987

[53] Dai S‐H , Wu Q‐C , Zhu R‐R , Wan X‐M , Zhou X‐L . Notch1 protects against myocardial ischaemia‐reperfusion injury via regulating mitochondrial fusion and function. J Cell Mol Med. 2020;24(5):3183‐3191. 31975567 10.1111/jcmm.14992 PMC7077547

[54] Okatan EN , Olgar Y , Tuncay E , Turan B . Azoramide improves mitochondrial dysfunction in palmitate‐induced insulin resistant H9c2 cells. Mol Cell Biochem. 2019;461(1–2):65‐72. 31327095 10.1007/s11010-019-03590-z

[55] Olmedo I , Pino G , Riquelme JA , et al. Inhibition of the proteasome preserves Mitofusin‐2 and mitochondrial integrity, protecting cardiomyocytes during ischemia‐reperfusion injury. Biochim Biophys Acta Mol Basis Dis. 2020;1866(5):165659. 31891806 10.1016/j.bbadis.2019.165659

[56] Hall AR , Burke N , Dongworth RK , et al. Hearts deficient in both Mfn1 and Mfn2 are protected against acute myocardial infarction. Cell Death Dis. 2016;7(5):e2238. 27228353 10.1038/cddis.2016.139 PMC4917668

[57] Tian F , Zhang Y . Overexpression of SERCA2a alleviates cardiac microvascular ischemic injury by suppressing Mfn2‐Mediated ER/Mitochondrial calcium tethering. Front Cell Dev Biol. 2021;9:636553. 33869181 10.3389/fcell.2021.636553 PMC8047138

[58] Zhang Y , Wang Y , Xu J , et al. Melatonin attenuates myocardial ischemia‐reperfusion injury via improving mitochondrial fusion/mitophagy and activating the AMPK‐OPA1 signaling pathways. J Pineal Res. 2019;66(2):e12542. 30516280 10.1111/jpi.12542

[59] Belenguer P , Pellegrini L . The dynamin GTPase OPA1: more than mitochondria? Biochim Biophys Acta. 2013;1833(1):176‐183. 22902477 10.1016/j.bbamcr.2012.08.004

[60] Rosselin M , Santo‐Domingo J , Bermont F , Giacomello M , Demaurex N . L‐OPA1 regulates mitoflash biogenesis independently from membrane fusion. EMBO Rep. 2017;18(3):451‐463. 28174208 10.15252/embr.201642931 PMC5331265

[61] Anand R , Wai T , Baker MJ , et al. The i‐AAA protease YME1L and OMA1 cleave OPA1 to balance mitochondrial fusion and fission. J Cell Biol. 2014;204(6):919‐929. 24616225 10.1083/jcb.201308006 PMC3998800

[62] Duvezin‐Caubet S , Jagasia R , Wagener J , et al. Proteolytic processing of OPA1 links mitochondrial dysfunction to alterations in mitochondrial morphology. J Biol Chem. 2006;281(49):37972‐37979. 17003040 10.1074/jbc.M606059200

[63] Baker MJ , Lampe PA , Stojanovski D , et al. Stress‐induced OMA1 activation and autocatalytic turnover regulate OPA1‐dependent mitochondrial dynamics. EMBO J. 2014;33(6):578‐593. 24550258 10.1002/embj.201386474 PMC3989652

[64] Twig G , Elorza A , Molina AJA , et al. Fission and selective fusion govern mitochondrial segregation and elimination by autophagy. EMBO J. 2008;27(2):433‐446. 18200046 10.1038/sj.emboj.7601963 PMC2234339

[65] Wai T , García‐Prieto J , Baker MJ , et al. Imbalanced OPA1 processing and mitochondrial fragmentation cause heart failure in mice. Science. 2015;350(6265):aad0116. 26785494 10.1126/science.aad0116

[66] Chen Y , Li S , Zhang Y , et al. The lncRNA Malat1 regulates microvascular function after myocardial infarction in mice via miR‐26b‐5p/Mfn1 axis‐mediated mitochondrial dynamics. Redox Biol. 2021;41:101910. 33667993 10.1016/j.redox.2021.101910 PMC7937833

[67] Li J , Li Y , Jiao J , et al. Mitofusin 1 is negatively regulated by microRNA 140 in cardiomyocyte apoptosis. Mol Cell Biol. 2014;34(10):1788‐1799. 24615014 10.1128/MCB.00774-13 PMC4019028

[68] Ferreira JCB , Campos JC , Qvit N , et al. A selective inhibitor of mitofusin 1‐βIIPKC association improves heart failure outcome in rats. Nat Commun. 2019;10(1):329. 30659190 10.1038/s41467-018-08276-6 PMC6338754

[69] Maneechote C , Palee S , Kerdphoo S , Jaiwongkam T , Chattipakorn SC , Chattipakorn N . Balancing mitochondrial dynamics via increasing mitochondrial fusion attenuates infarct size and left ventricular dysfunction in rats with cardiac ischemia/reperfusion injury. Clin Sci (Lond). 2019;133(3):497‐513. 30705107 10.1042/CS20190014

[70] Kang SWS , Haydar G , Taniane C , et al. AMPK activation prevents and reverses drug‐induced mitochondrial and hepatocyte injury by promoting mitochondrial fusion and function. PLoS One. 2016;11(10):e0165638. 27792760 10.1371/journal.pone.0165638 PMC5085033

[71] Liu D , Ma Z , Di S , et al. AMPK/PGC1alpha activation by melatonin attenuates acute doxorubicin cardiotoxicity via alleviating mitochondrial oxidative damage and apoptosis. Free Radic Biol Med. 2018;129:59‐72. 30172748 10.1016/j.freeradbiomed.2018.08.032

[72] Xue W , Wang X , Tang H , et al. Vitexin attenuates myocardial ischemia/reperfusion injury in rats by regulating mitochondrial dysfunction induced by mitochondrial dynamics imbalance. Biomed Pharmacother. 2020;124:109849. 31972356 10.1016/j.biopha.2020.109849

[73] Xin T , Lu C . Irisin activates Opa1‐induced mitophagy to protect cardiomyocytes against apoptosis following myocardial infarction. Aging (Albany NY). 2020;12(5):4474‐4488. 32155590 10.18632/aging.102899 PMC7093202

[74] Meeusen SL , Nunnari J . How mitochondria fuse. Curr Opin Cell Biol. 2005;17(4):389‐394. 15975776 10.1016/j.ceb.2005.06.014

[75] Gao J , Zhao L , Wang J , et al. C‐Phycocyanin ameliorates mitochondrial fission and fusion dynamics in ischemic cardiomyocyte damage. Front Pharmacol. 2019;10:733. 31316386 10.3389/fphar.2019.00733 PMC6611522

[76] Zhao D , Sun Y , Tan Y , et al. Short‐duration swimming exercise after myocardial infarction attenuates cardiac dysfunction and regulates mitochondrial quality control in aged mice. Oxid Med Cell Longev. 2018;2018:4079041. 29849892 10.1155/2018/4079041 PMC5925211

[77] Naruse G , Kanamori H , Yoshida A , et al. The intestine responds to heart failure by enhanced mitochondrial fusion through glucagon‐like peptide‐1 signalling. Cardiovasc Res. 2019;115(13):1873‐1885. 30629149 10.1093/cvr/cvz002

[78] Ney PA . Mitochondrial autophagy: Origins, significance, and role of BNIP3 and NIX. Biochim Biophys Acta. 2015;1853(10):2775‐2783. 25753537 10.1016/j.bbamcr.2015.02.022

[79] Lemasters JJ . Selective mitochondrial autophagy, or mitophagy, as a targeted defense against oxidative stress, mitochondrial dysfunction, and aging. Rejuvenation Res. 2005;8(1):3‐5. 15798367 10.1089/rej.2005.8.3

[80] Yao LI , Chen H , Wu Q , Xie K . Hydrogen‐rich saline alleviates inflammation and apoptosis in myocardial I/R injury via PINK‐mediated autophagy. Int J Mol Med. 2019;44(3):1048‐1062. 31524220 10.3892/ijmm.2019.4264 PMC6657957

[81] Koyano F , Okatsu K , Kosako H , et al. Ubiquitin is phosphorylated by PINK1 to activate parkin. Nature. 2014;510(7503):162‐166. 24784582 10.1038/nature13392

[82] Bingol B , Sheng M . Mechanisms of mitophagy: PINK1, Parkin, USP30 and beyond. Free Radic Biol Med. 2016;100:210‐222. 27094585 10.1016/j.freeradbiomed.2016.04.015

[83] Wei Y , Chiang W‐C , Sumpter R , Mishra P , Levine B . Prohibitin 2 Is an inner mitochondrial membrane mitophagy receptor. Cell. 2017;168(1–2):224‐238.e10. 28017329 10.1016/j.cell.2016.11.042 PMC5235968

[84] Xu G , Shen H , Nibona E , et al. Fundc1 is necessary for proper body axis formation during embryogenesis in zebrafish. Sci Rep. 2019;9(1):18910. 31827208 10.1038/s41598-019-55415-0 PMC6906497

[85] Vasagiri N , Kutala VK . Structure, function, and epigenetic regulation of BNIP3: a pathophysiological relevance. Mol Biol Rep. 2014;41(11):7705‐7714. 25096512 10.1007/s11033-014-3664-x

[86] Liu L , Feng D , Chen G , et al. Mitochondrial outer‐membrane protein FUNDC1 mediates hypoxia‐induced mitophagy in mammalian cells. Nat Cell Biol. 2012;14(2):177‐185. 22267086 10.1038/ncb2422

[87] Zhang W , Siraj S , Zhang R , Chen Q . Mitophagy receptor FUNDC1 regulates mitochondrial homeostasis and protects the heart from I/R injury. Autophagy. 2017;13(6):1080‐1081. 28323531 10.1080/15548627.2017.1300224 PMC5486361

[88] Zhou H , Zhu P , Wang J , Zhu H , Ren J , Chen Y . Pathogenesis of cardiac ischemia reperfusion injury is associated with CK2alpha‐disturbed mitochondrial homeostasis via suppression of FUNDC1‐related mitophagy. Cell Death Differ. 2018;25(6):1080‐1093. 29540794 10.1038/s41418-018-0086-7 PMC5988750

[89] Yu W , Xu M , Zhang T , Zhang Q , Zou C . Mst1 promotes cardiac ischemia‐reperfusion injury by inhibiting the ERK‐CREB pathway and repressing FUNDC1‐mediated mitophagy. J Physiol Sci. 2019;69(1):113‐127. 29961191 10.1007/s12576-018-0627-3 PMC10717665

[90] Lee Y , Lee H‐Y , Hanna RA , Gustafsson ÅB . Mitochondrial autophagy by Bnip3 involves Drp1‐mediated mitochondrial fission and recruitment of Parkin in cardiac myocytes. Am J Physiol Heart Circ Physiol. 2011;301(5):H1924‐H1931. 21890690 10.1152/ajpheart.00368.2011 PMC3213962

[91] Zhang Y , Liu D , Hu H , Zhang P , Xie R , Cui W . HIF‐1alpha/BNIP3 signaling pathway‐induced‐autophagy plays protective role during myocardial ischemia‐reperfusion injury. Biomed Pharmacother. 2019;120:109464. 31590128 10.1016/j.biopha.2019.109464

[92] Song X , Li T . Ripk3 mediates cardiomyocyte necrosis through targeting mitochondria and the JNK‐Bnip3 pathway under hypoxia‐reoxygenation injury. J Recept Signal Transduct Res. 2019;39(4):331‐340. 31658855 10.1080/10799893.2019.1676259

[93] Li Y , Ren S , Xia J , Wei Y , Xi Y . EIF4A3‐Induced circ‐BNIP3 aggravated hypoxia‐induced injury of H9c2 cells by targeting miR‐27a‐3p/BNIP3. Mol Ther Nucleic Acids. 2020;19:533‐545. 31923741 10.1016/j.omtn.2019.11.017 PMC6951839

[94] Cheng N , Wang MY , Wu YB , et al. Circular RNA POSTN promotes myocardial infarction‐induced myocardial injury and cardiac remodeling by regulating miR‐96‐5p/BNIP3 Axis. Front Cell Dev Biol. 2020;8:618574. 33681183 10.3389/fcell.2020.618574 PMC7930329

[95] Zhu P , Wan K , Yin M , et al. RIPK3 induces cardiomyocyte necroptosis via inhibition of AMPK‐Parkin‐Mitophagy in cardiac remodelling after myocardial infarction. Oxid Med Cell Longev. 2021;2021:6635955. 33854696 10.1155/2021/6635955 PMC8019651

[96] Feng L , Zhou D , Luo C , et al. Ferulic acid attenuates Hypoxia/Reoxygenation injury by suppressing mitophagy through the PINK1/Parkin signaling pathway in H9c2 cells. Front Pharmacol. 2020;11:103. 32161543 10.3389/fphar.2020.00103 PMC7052384

[97] Feng Y , Madungwe NB , da Cruz Junho CV , Bopassa JC . Activation of G protein‐coupled oestrogen receptor 1 at the onset of reperfusion protects the myocardium against ischemia/reperfusion injury by reducing mitochondrial dysfunction and mitophagy. Br J Pharmacol. 2017;174(23):4329‐4344. 28906548 10.1111/bph.14033 PMC5715577

[98] Gustafsson AB . Bnip3 as a dual regulator of mitochondrial turnover and cell death in the myocardium. Pediatr Cardiol. 2011;32(3):267‐274. 21210091 10.1007/s00246-010-9876-5 PMC3051075

[99] Li Y , Liu Z , Zhang Y , et al. PEDF protects cardiomyocytes by promoting FUNDC1mediated mitophagy via PEDF‐R under hypoxic condition. Int J Mol Med. 2018;41(6):3394‐3404. 29512692 10.3892/ijmm.2018.3536 PMC5881750

[100] Zhou H , Li D , Zhu P , et al. Melatonin suppresses platelet activation and function against cardiac ischemia/reperfusion injury via PPARgamma/FUNDC1/mitophagy pathways. J Pineal Res. 2017;63(4):e12438. 10.1111/jpi.12438 28749565

[101] Yang L , Xie P , Wu J , et al. Deferoxamine treatment combined with sevoflurane postconditioning attenuates myocardial ischemia‐reperfusion injury by restoring HIF‐1/BNIP3‐Mediated mitochondrial autophagy in GK Rats. Front Pharmacol. 2020;11:6. 32140105 10.3389/fphar.2020.00006 PMC7042377

[102] Liu XW , Lu MK , Zhong HT , Wang LH , Fu YP . Panax Notoginseng Saponins attenuate myocardial ischemia‐reperfusion injury through the HIF‐1alpha/BNIP3 pathway of autophagy. J Cardiovasc Pharmacol. 2019;73(2):92‐99. 30531436 10.1097/FJC.0000000000000640

[103] Shin S , Choi J‐W , Moon H , et al. Simultaneous suppression of multiple programmed cell death pathways by miRNA‐105 in cardiac ischemic injury. Mol Ther Nucleic Acids. 2019;14:438‐449. 30743213 10.1016/j.omtn.2018.12.015 PMC6369328

[104] Yeang C , Hasanally D , Que X , et al. Reduction of myocardial ischaemia‐reperfusion injury by inactivating oxidized phospholipids. Cardiovasc Res. 2019;115(1):179‐189. 29850765 10.1093/cvr/cvy136 PMC6302283

[105] Huang Z , Ye B , Dai Z , et al. Curcumin inhibits autophagy and apoptosis in hypoxia/reoxygenation‐induced myocytes. Mol Med Rep. 2015;11(6):4678‐4684. 25673156 10.3892/mmr.2015.3322

[106] Liu S , Ai Q , Feng K , Li Y , Liu X . The cardioprotective effect of dihydromyricetin prevents ischemia‐reperfusion‐induced apoptosis in vivo and in vitro via the PI3K/Akt and HIF‐1alpha signaling pathways. Apoptosis. 2016;21(12):1366‐1385. 27738772 10.1007/s10495-016-1306-6

[107] Yu Z , Gong X , Yu Y , et al. The mechanical effects of CRT promoting autophagy via mitochondrial calcium uniporter down‐regulation and mitochondrial dynamics alteration. J Cell Mol Med. 2019;23(6):3833‐3842. 30938090 10.1111/jcmm.14227 PMC6533471

[108] Zhu NA , Cao X , Hao P , et al. Berberine attenuates mitochondrial dysfunction by inducing autophagic flux in myocardial hypoxia/reoxygenation injury. Cell Stress Chaperones. 2020;25(3):417‐426. 32088907 10.1007/s12192-020-01081-5 PMC7193011

[109] Wu NN , Tian H , Chen P , Wang D , Ren J , Zhang Y . Physical exercise and selective autophagy: benefit and risk on cardiovascular health. Cells. 2019;8(11):1436. 10.3390/cells8111436 PMC6912418 31739509

[110] Li J‐Y , Pan S‐S , Wang J‐Y , Lu J . Changes in autophagy levels in rat myocardium during exercise preconditioning‐initiated cardioprotective effects. Int Heart J. 2019;60(2):419‐428. 30745541 10.1536/ihj.18-310

[111] Yuan Y , Pan SS . Parkin mediates mitophagy to participate in cardioprotection induced by late exercise preconditioning but Bnip3 does not. J Cardiovasc Pharmacol. 2018;71(5):303‐316. 29538088 10.1097/FJC.0000000000000572

[112] Yuan Y , Pan SS , Wan DF , Lu J , Huang Y . H2O2 Signaling‐Triggered PI3K mediates mitochondrial protection to participate in early cardioprotection by exercise preconditioning. Oxid Med Cell Longev. 2018;2018:1916841. 30147831 10.1155/2018/1916841 PMC6083504

[113] Czegledi A , Tosaki A , Gyongyosi A , Zilinyi R , Tosaki A , Lekli I . Electrically‐Induced ventricular fibrillation alters cardiovascular function and expression of apoptotic and autophagic proteins in rat hearts. Int J Mol Sci. 2019;20(7):1628. 10.3390/ijms20071628 PMC6479527 30986903

[114] Liu J , Yan W , Zhao X , et al. Sirt3 attenuates post‐infarction cardiac injury via inhibiting mitochondrial fission and normalization of AMPK‐Drp1 pathways. Cell Signal. 2019;53:1‐13. 30219671 10.1016/j.cellsig.2018.09.009

[115] East DA , Campanella M . Mitophagy and the therapeutic clearance of damaged mitochondria for neuroprotection. Int J Biochem Cell Biol. 2016;79:382‐387. 27586258 10.1016/j.biocel.2016.08.019

[116] East DA , Fagiani F , Crosby J , et al. PMI: a DeltaPsim independent pharmacological regulator of mitophagy. Chem Biol. 2014;21(11):1585‐1596. 25455860 10.1016/j.chembiol.2014.09.019 PMC4245710

[117] Park CW , Hong SM , Kim E‐S , et al. BNIP3 is degraded by ULK1‐dependent autophagy via MTORC1 and AMPK. Autophagy. 2013;9(3):345‐360. 23291726 10.4161/auto.23072 PMC3590255

